# Clinical evaluation of urine laminin‐γ2 monomer as a potent biomarker for non‐muscle invasive bladder cancer

**DOI:** 10.1002/cam4.5087

**Published:** 2022-08-04

**Authors:** Takashi Karashima, Susumu Umemoto, Takeshi Kishida, Kimito Osaka, Masatoshi Nakagawa, Eisaku Yoshida, Toru Yoshimura, Masahiko Sakaguchi, Hiroyuki Nishimoto, Mami Tai, Keiji Inoue, Motoharu Seiki, Naohiko Koshikawa, Taro Shuin

**Affiliations:** ^1^ Department of Urology Kochi Medical School Nankoku Japan; ^2^ Department of Urology Kanagawa Cancer Center Yokohama Japan; ^3^ Diagnostic Division Abbott Japan LLC Chiba Japan; ^4^ Integrated Center for Advanced Medical Technologies Kochi Medical School Nankoku Japan; ^5^ Division of Cancer Prevention and Control Kanagawa Cancer Center Research Institute Yokohama Japan; ^6^ School of Medicine Kanazawa University Kanazawa Japan; ^7^ Institute of Medical Science University of Tokyo Tokyo Japan; ^8^ Division of Cancer Cell Research Kanagawa Cancer Center Research Institute Yokohama Japan

**Keywords:** biomarker, bladder cancer, laminin‐γ2, non‐muscle‐invasive bladder cancer, urine laminin‐γ2 monomer

## Abstract

**Background:**

To evaluate whether urine laminin‐γ2 monomer (Ln‐γ2m) offers a useful biomarker for patients with non‐muscle‐invasive bladder cancer (NMIBC).

**Methods:**

Participants comprised 297 patients, including 111 patients with NMIBC, 136 patients with benign genitourinary disease (BD) and 50 healthy donors (HD). Urine Ln‐γ2m was prospectively measured and accuracy was analyzed. Receiver operating characteristic (ROC) curves were determined and area under the ROC curve (AUC) was calculated for urine Ln‐γ2m, and compared to those of traditional urine tumor markers such as nuclear matrix protein 22 (NMP22), bladder tumor antigen (BTA) and cytology. The net benefits of combining urine markers were analyzed by decision curve analysis.

**Results:**

Mean urine Ln‐γ2m was significantly higher in NMIBC than in BD or HD. The AUC for urine Ln‐γ2m was significantly higher than those for urine NMP22, BTA or cytology when comparing NMIBC with HD. In patients with low‐grade NMIBC, the AUC for urine Ln‐γ2m was higher than the AUCs for NMP22, BTA or cytology. A net benefit of combined examination using urine Ln‐γ2m/uCRN with NMP22 was demonstrated.

**Conclusion:**

These results suggest urine Ln‐γ2m as a potentially useful biomarker for NMIBC, particularly in cases of low‐grade cancer.

## Graphical abstract

To evaluate whether measurement of urine laminin‐γ2 monomer (Ln‐γ2m) offers a useful biomarker for patients with non‐muscle invasive bladder cancer (NMIBC). The measurement of urine Ln‐γ2m offers a potentially useful biomarker for NMIBC, particularly in cases of low‐grade cancer.

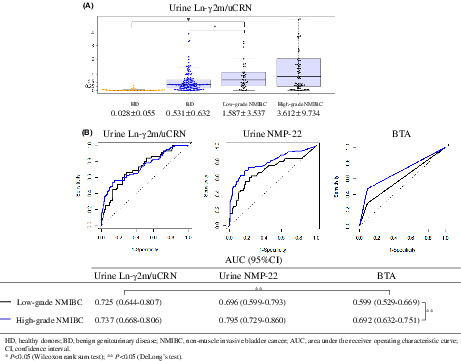

## 1 INTRODUCTION

The age‐standardized annual incidence of bladder cancer in Japan is 7.2/100,000 population/year. The incidence of bladder cancer appears to be increasing with the continued aging of the population.^1^ Around 70% of bladder cancers are non‐muscle‐invasive bladder cancers (NMIBCs), which show good prognosis with endoscopic surgery.^2^ However, the clinical features of bladder cancer include frequent recurrence with temporal and spatial multiplicity after complete endoscopic resection of visible lesions.^3^ Patients with NMIBC must therefore undergo repeated invasive examinations, including cystoscopy, followed by repeated endoscopic surgeries for recurrences. This represents a major issue for patients with NMIBC, particularly for Japanese patients, given the long life expectancy in Japan.

Urine cytology, nuclear matrix protein 22 (NMP22), and bladder tumor antigen (BTA) are currently available as urine tumor markers for diagnosing bladder cancer in Japan. Urine cytology is the most common non‐invasive examination for bladder pathologies. This modality represents an accurate examination for diagnosing high‐grade muscle‐invasive bladder cancer (MIBC), but offers insufficient sensitivity for diagnosing NMIBC. As a result, most patients with NMIBC require repeated cystoscopies for the diagnosis of recurrence. NMP22 is a nuclear protein selectively expressed by cancers of the bladder and upper urinary tract, whereas BTA is produced by the digestion of extracellular matrix in the basement membranes of bladder and upper urinary tract cancers.^4^ The sensitivities of NMP22 and BTA are 58%–69% and 64%–65%, respectively, and the specificities are 77%–88% and 74%–77%, respectively.^4^ Neither test is in routine use for diagnosing NMIBC in Japan, because the false‐positive rates for NMP22 and BTA are high in non‐BC patients with hematuria, urinary infections, and urolithiasis.^4^, ^5^ Development of novel, non‐invasive, highly specific urine tumor markers is thus sorely needed for the routine monitoring of patients with NMIBC.

Laminin (Ln)‐332 comprises three polypeptides encoded by different genes, namely Ln‐α3, −β3, and ‐γ2, and acts as an adhesive molecule in the basement membrane of normal epithelia.^6^ Ln‐γ2 expressed as a monomer in invasive tumor cells and tissues has recently been reported to play a crucial role in the malignant progression of tumor cell processes such as growth, survival, motility, invasion, and metastasis.^7^ We have previously reported that Ln‐γ2 monomer (Ln‐γ2m) was highly expressed at the invasive front of bladder cancer tissue according to immunohistochemical staining (Figure S1), and was detectable in the urine of patients with bladder cancer by Western blot and enzyme‐linked immunosorbent assay.^8^ We developed a fully automated chemiluminescent immunoassay using a specific monoclonal antibody against Ln‐γ2m, providing highly accurate measurements of urine Ln‐γ2m in patients with NMIBC rather than MIBC.^9^ As those studies were basic retrospective studies, a large, prospective study examining the utility of urine Ln‐γ2m as a biomarker for patients with NMIBC was needed.

This study prospectively analyzed whether urine Ln‐γ2m levels as determined by chemiluminescent immunoassay offered better diagnostic accuracy than urine levels of NMP22 or BTA, or urine cytology in patients with NMIBC, patients with benign genitourinary disease (BD), and healthy donors (HD).

## 2 METHODS

### 2.1 Study design and population

This multicenter, prospective study involved Kochi Medical School Hospital and Kanagawa Cancer Center. Between February 2016 and May 2018, patients with diagnoses of NMIBC (*n* = 111) or BD (*n* = 136) identified at Kochi Medical School or Kanagawa Cancer Center, and HD (*n* = 50) at Kochi Medical School were enrolled in the study. The full eligibility criteria are listed in Appendix S1 (Eligibility Criteria**)**. All participants were Japanese and all patients with NMIBC were definitively diagnosed based on the histopathological findings of specimens obtained from transurethral resection of bladder tumor. The sample size was calculated for multivariable logistic regression analysis in this study.^10^ HD showed neither medical problems nor allergies. All patients and HD provided written informed consent for participation in this investigation, and the study protocol was approved by the review boards of both participating institutions (Kochi Medical School Hospital: approval no. 27–63; Kanagawa Cancer Center: approval no. 27–21). This study complied with the Japanese ethical guidelines for medical and health research involving human subjects and the Declaration of Helsinki.^11^ Median ages at acquisition of consent for NMIBC, BD, and HD were 73 years, 70 years, and 24 years (interquartile ranges, 67–79 years, 63–75 years, and 23–25 years), respectively (Table 1). No occurrence of bladder cancer was observed among HD and BD patients during the observation period.

**TABLE 1:** Characteristics of evaluated patients with non‐muscle invasive bladder cancer, patients with benign genitourinary disease and healthy donors

Group	Age (range) or *n* (%)
Non‐muscle invasive bladder cancer	111
Mean age	73 (67–79) years
Sex	
Male	88 (79)
Female	23 (21)
pT stage	
Ta	74
T1	33
Tis	4
Grade	
Low	45 (41)
High	66 (59)
Benign genitourinary disease	136
Mean age	70 (63–75) years
BPH	49 (36)
OAB	22 (16)
Inflammation	17 (13)
Urolithiasis	16 (12)
Others	32 (23)
Healthy donors	50
Mean age	24 (23–25) years

Abbreviations: BPH, benign prostatic hyperplasia; OAB, overactive bladder.

### 2.2 Data management

All clinical and examination data were sent to the Data Center at the Integrated Center for Advanced Medical Technologies, Kochi Medical School using an Electronic Data Capture system. All data were managed by the data manager in the Data Center, then analyzed by an independent statistician.

### 2.3 Clinical urine specimens

Urine specimens were centrifuged for 5 min at 200×*g* after collection from patients and HD, then stored at −80°C for 1–6 months. Specimens (250 μl) were then subjected to automated chemiluminescent immunoassay after centrifugation for 10 min at 15,000 rpm at 4°C. Values of urine Ln‐γ2m were normalized to urine creatinine (uCRN) concentrations, as described in detail below.

### 2.4 Detection of urine Ln‐γ2m by chemiluminescent immunoassay

Anti‐Ln‐γ2m monoclonal antibody (2H2 mAb) was established in our laboratory.^12^ The recombinant Ln‐γ2 domain III (DIII) protein (amino acids 383–608) was used for rabbit immunization to generate Ln‐γ2 polyclonal antibody. A two‐step sandwich assay using the 2H2 mAb and Ln‐γ2 polyclonal antibody was performed for the fully automated chemiluminescent immunoassay using the ARCHITECT system (Abbott Laboratories). The measurement range for Ln‐γ2m was 0–20,000 pg/ml.^9^

### 2.5 Measurement of urine creatinine, NMP22 and BTA

Measurements of all examinations including urine creatinine, NMP22 and BTA were performed by SRL Inc. Concentrations of uCRN were measured using a colorimetric assay and the Determiner‐L creatinine system (Kyowa Medex). Urine Ln‐γ2m concentrations were divided by uCRN, yielding the urine Ln‐γ2m/uCRN (ng/g·CRN × 100).^8^ Urine NMP22 was measured by sandwich enzyme‐linked immunosorbent assay, using the qualitative Alere NMP22 test (Alere Medical), with a cut‐off value of 12.0 U/ml.^13^ Positive or negative status of urine BTA was determined by latex agglutination assay for qualitative detection in urine (V‐BTA test; Polymedco).^14^

### 2.6 Urine cytology

Urine cytology was examined by SRL Inc. using an evaluation method adopted by many medical institutions in Japan. Urine cytology was sorted into 7 classifications: (1) absence of atypical or abnormal cells; (2) atypical cytology, but no evidence of malignancy; (3a) probable benign atypia; (3) cytology suggestive of, but not conclusive for malignancy; (3b) suspected malignancy; (4) cytology strongly suggestive of malignancy; and (5) cytology conclusive for malignancy.^15^ In the present study, classes 4 and 5 were grouped together as positive for malignancy.

### 2.7 Statistical analysis

Statistical analysis was performed using R version 3.3.3 (The R Foundation, Vienna, Austria). The Wilcoxon rank sum test and Fisher's exact test were used for comparisons of two groups. The pROC and dcurves packages (The R Foundation) were used for analysis of receiver operating characteristic (ROC) curves and decision curve analysis (DCA), respectively. The predictive performances of diagnostic markers including urine Ln‐γ2m/uCRN, NMP22, BTA and cytology were evaluated using ROC analysis, area under the ROC curve (AUC), and corresponding 95% confidence intervals (CIs).^16^ Statistical differences between two correlated ROC curves were analyzed using DeLong's test. The diagnostic values of diagnostic markers were evaluated based on AUCs, with sensitivity and specificity evaluated at three cut‐off values, calculated as: maximum Youden index^17^; maximum sensitivity with specificity ≥80%; and maximum specificity with sensitivity ≥80%. Exploratory clinical consequences of combination with urine markers were analyzed by DCA, as: Net benefit (*v*) = TP (*v*) − {*v*/(1 − *v*)} FP (*v*), where *v* is threshold probability, and TP and FP are the true‐ and false‐positive rates, respectively.^18^

## 3 RESULTS

### 3.1 Values of urine Ln‐γ2m/uCRN, NMP22, BTA, and cytology for HD, BD, and NMIBC

**FIGURE 1:**
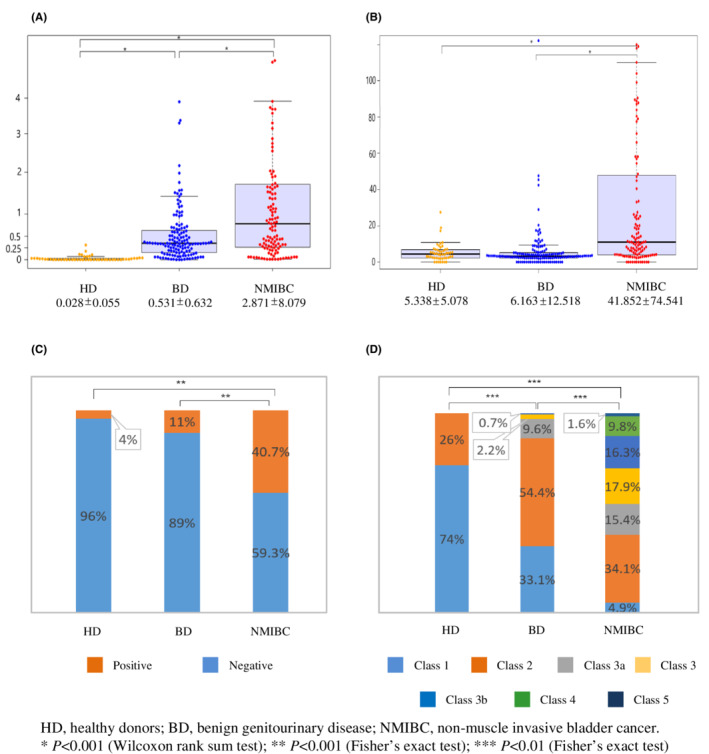
Dot plots of urine Ln‐γ2m/uCRN (A) and NMP22 (B), and bar graph of urine BTA (C), and cytology (D) for healthy donors (HD), patients with benign genitourinary disease (BD), and patients with non‐muscle invasive bladder cancer (NMIBC). Mean and standard deviation of urine Ln‐γ2m/uCRN and NMP22, and percentage frequencies of positive and negative results for urine BTA, and percentage frequencies of urine cytology classifications are shown.

Values of urine Ln‐γ2m/uCRN, NMP22, BTA, and cytology obtained from HD, BD, and NMIBC were analyzed. The value of urine Ln‐γ2m/uCRN was significantly higher for NMIBC (2.871 ± 8.079 ng/g·CRN × 100) than for HD (0.028 ± 0.055) or BD (0.531 ± 0.632). The value of urine Ln‐γ2m/uCRN was significantly higher in BD than in HD (*p* < 0.001; Figure 1A). Urine NMP22, BTA and cytology were also analyzed (Figure 1B–D). The value of urine NMP22 was significantly higher for NMIBC (41.852 ± 74.541 U/ml) than for HD (5.338 ± 5.078) or BD (6.163 ± 12.518) (*p* < 0.001). In contrast, the value of urine NMP22 in HD and BD did not show any significant difference (Figure 1B). Using a cut‐off value for urine BTA of 20 μg/ml, the positive ratio for patients with NMIBC was 40.7%, significantly higher than in HD (4%) or BD (11%) (*p* < 0.001, Figure 1C). Urine cytology of NMIBC was class 1 in 4.9%, class 2 in 34.1%, class 3a in 15.4%, class 3 in 17.9%, class 3b in 16.3%, class 4 in 9.8%, and class 5 in 1.6%. Although the specificity of urine cytology was 100%, sensitivity was only 11.4% when class 4 or 5 cytology was grouped together as positive for malignancy. Each group showed a significant difference (*p* < 0.001 each).

### 3.2 Comparative diagnostic accuracies of urine Ln‐γ2m/CRN, NMP22, BTA, and cytology

**FIGURE 2:**
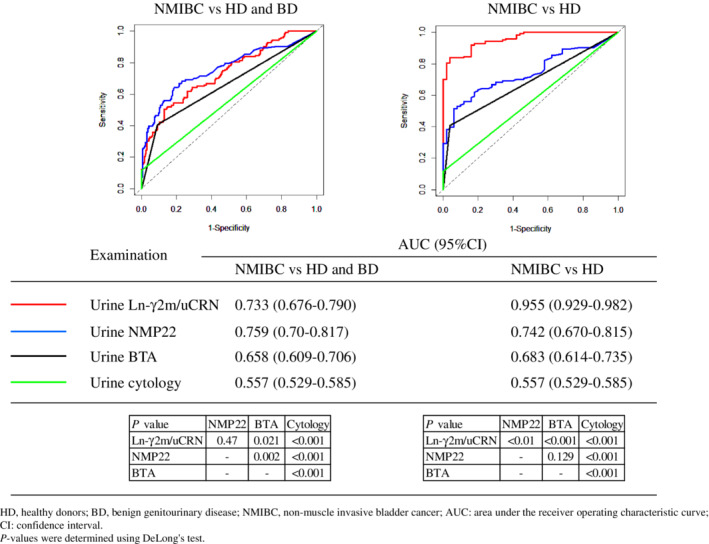
Receiver operating characteristic curves for urine Ln‐γ2m/uCRN, NMP22, BTA, and cytology in patients with non‐muscle invasive bladder cancer (NMIBC) compared to healthy donors (HD) and patients with benign genitourinary disease (BD, left panel), or NMIBC compared to HD (right panel). Area under the receiver operating characteristic curve (AUC) for each examination is provided. *p*‐values for comparisons of each AUC are provided at the bottom of the figure.

We compared diagnostic accuracies between urine Ln‐γ2m/uCRN, NMP22, BTA, and cytology in patients with NMIBC, HD or BD using ROC curve analysis (Figure 2). For urine cytology, we judged classes 1, 2, 3a, 3 and 3b as negative for malignancy, and classes 4 and 5 were judged as positive for malignancy. When compared ROC curves of urine Ln‐γ2m/uCRN, NMP22, BTA, and cytology in patients with NMIBC compared to HD and BD, AUCs were 0.733, 0.759, 0.658, and 0.557, respectively. When we compared ROC curves in patients with NMIBC versus HD, AUCs of urine Ln‐γ2m/uCRN, NMP22, BTA, and cytology were 0.955, 0.742, 0.683, and 0.557, respectively. The *p*‐values for comparisons of each examination are shown in Figure 2.

Table 2 shows sensitivities, specificities and accuracies of urine Ln‐γ2m/uCRN and NMP22 at 3 cut‐off points setting the sensitivity‐dominant criterion (criterion A), specificity‐dominant criterion (criterion B), and maximum Youden index (criterion C). When the cut‐off value for the urine Ln‐γ2m/uCRN was 0.146 (ng/g·CRN × 100) at criterion A, sensitivity, specificity, and accuracy were 81.3%, 44.1%, and 58.9%, respectively. When the cut‐off value of urine Ln‐γ2m/uCRN was 0.778 (ng/g·CRN × 100) at criterion B, sensitivity, specificity, and accuracy were 50.4%, 87.1%, and 72.5%, respectively. When the cut‐off value of urine Ln‐γ2m/uCRN was 0.628 (ng/g·CRN × 100) at criterion C, sensitivity, specificity and accuracy were 54.5%, 81.7% and 70.9%, respectively. When the cut‐off value of urine NMP22 was 3.35 U/ml at criterion A, sensitivity, specificity, and accuracy were 80.5%, 47.8%, and 60.8%, respectively. When the cut‐off value of urine NMP22 was 7.15 U/ml at criterion B, sensitivity, specificity, and accuracy were 64.2%, 81.2%, and 74.4%, respectively. The cut‐off value based on maximum Youden index (criterion C) was the same as Criterion B, and sensitivity, specificity, and accuracy were the same. The accuracies for urine Ln‐γ2m/uCRN and NMP22 were equivalent with cut‐off values set using the sensitivity‐dominant criterion, specificity‐dominant criterion, and maximum Youden index.

**TABLE 2:** Accuracy of markers in patients with non‐muscle invasive bladder cancer, patients with benign genitourinary disease and healthy donors

Marker	Cut‐off	Sensitivity (95% CI)	Specificity (95% CI)	Accuracy (95% CI)
Urine Ln‐γ2m/uCRN	ng/g·CRN × 100			
Criterion A	0.146	81.3 (73.3–87.8)	44.1 (36.8–51.5)	58.9 (53.2–64.4)
Criterion B	0.778	50.4 (41.2–59.5)	87.1 (81.4–91.6)	72.5 (67.2–77.4)
Criterion C	0.628	54.5 (45.2–63.5)	81.7 (75.4–87.0)	70.9 (65.5–75.9)
Urine NMP22	U/ml			
Criterion A	3.35	80.5 (72.4–87.1)	47.8 (40.5–55.3)	60.8 (55.2–66.3)
Criterion B	7.15	64.2 (55.1–72.7)	81.2 (74.8–86.5)	74.4 (69.2–79.2)
Criterion C	7.15	64.2 (55.1–72.7)	81.2 (74.8–86.5)	74.4 (69.2–79.2)

Abbreviations: CI, confidence interval; Ln‐γ2m/uCRN, laminin‐γ2 monomer/urine creatinine; NMP22, nuclear matrix protein 22.

### 3.3 Diagnostic accuracy of urine Ln‐γ2m/uCRN in patients with low‐ and high‐grade NMIBC

**FIGURE 3:**
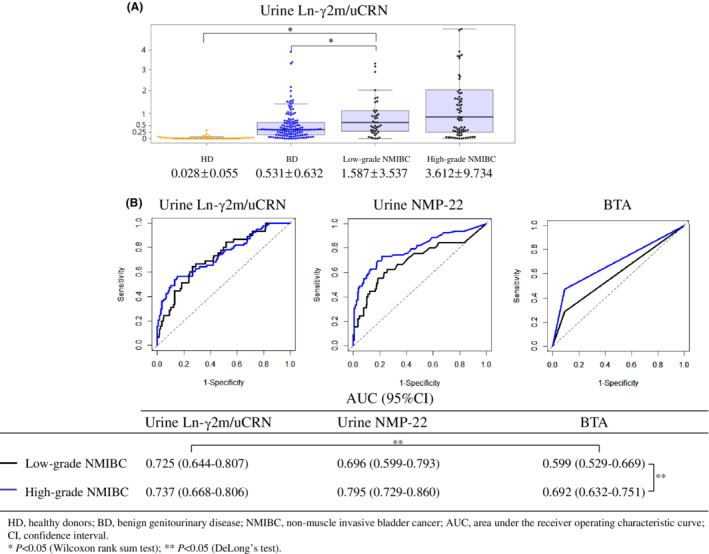
(A) Dot plots of urine Ln‐γ2m/uCRN for healthy donors (HD), patients with benign genitourinary disease (BD), and patients with low‐ and high‐grade non‐muscle‐invasive bladder cancer (NMIBC). (B) Receiver operating characteristic (ROC) curve of urine Ln‐γ2m/uCRN and NMP22 in patients with low‐grade NMIBC compared to HD and BD. Areas under the ROC curves (AUCs) for each examination are provided at the bottom of the figure. Mean and standard deviation for urine Ln‐γ2m/uCRN in HD, BD, and low‐ and high‐grade NMIBC are 0.028 ± 0.055, 0.531 ± 0.632, 1.587 ± 3.537 and 3.612 ± 9.734, respectively. AUCs for urine Ln‐γ2m/uCRN NMP22 and BTA in patients with low‐grade NMIBC are 0.725, 0.696 and 0.599, respectively. AUCs for urine Ln‐γ2m/uCRN, NMP22, and BTA in patients with high‐grade NMIBC are 0.737, 0.795, and 0.692, respectively.

We evaluated values of urine Ln‐γ2m/uCRN separately for low‐ or high‐grade NMIBC (Figure 3A). Mean urine Ln‐γ2m/uCRN for even low‐grade NMIBC (1.587 ± 3.537 ng/g·CRN × 100) was still significantly higher than those of either HD (0.028 ± 0.055 ng/g·CRN × 100; *p* < 0.05) or BD (0.531 ± 0.632 ng/g·CRN × 100; *p* < 0.05). We also separately evaluated the ROC curves of patients with low‐ and high‐grade NMIBC in examinations of urine Ln‐γ2m/uCRN, NMP22 and BTA. AUCs of urine BTA in low‐ and high‐grade NMIBC were 0.599 and 0.692, respectively, showing a significant difference (*p* < 0.05). In low‐grade NMIBC, the AUCs of urine Ln‐γ2m/uCRN were significantly higher than that of urine BTA.

### 3.4 DCA of combining urine Ln‐γ2m/uCRN and NMP22

**FIGURE 4:**
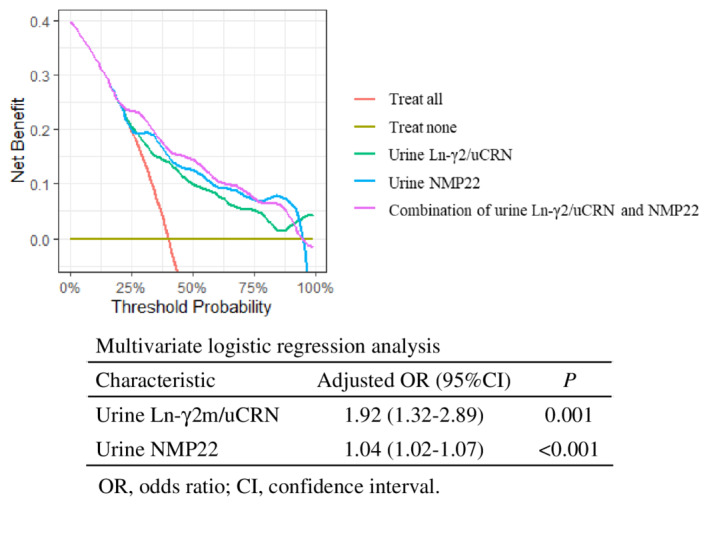
Adjusted odds ratios, 95% CIs, and *p*‐values for measurement of urine Ln‐γ2m/uCRN and NMP22 are 1.92; 1.32–2.89; *p* = 0.001 and 1.04; 1.02–1.07; *p* < 0.001 by multivariate logistic regression analysis. The decision curve analysis shows urine Ln‐γ2/uCRN (green), NMP22 (blue) and the combination (pink). The curve for the combination is located above each individual examination.

Measurement of both urine Ln‐γ2m/uCRN (adjusted odds ratio 1.92, 95% CI 1.32–2.89; *p* = 0.001) and NMP22 (adjusted odds ratio 1.04, 95% CI 1.02–1.07; *p* < 0.001) was demonstrated as an effective examination for detecting NMIBC by multivariate logistic regression analysis. We used DCA to evaluate whether combined measurement with urine Ln‐γ2m/uCRN and NMP22 had potential clinical benefit. The curve for combined examination was located above each single examination (Figure 4).

## 4 DISCUSSION

A retrospective study by Kamada et al. reported that measurement of the urine Ln‐γ2 chain showed significantly higher odds ratios for detection of NMIBC in logistic regression analyses, compared to urine NMP22 and BTA.^8^ The present prospective study analyzed the diagnostic accuracy of urine Ln‐γ2m in patients with NMIBC and demonstrated that the AUC for urine Ln‐γ2m/uCRN was equivalent to that for urine NMP22 and higher than those for urine BTA and cytology, when NMIBC was compared to BD and HD.

In the literature, age >60 years, smoking, urinary calculi, presence of leukocytes, urinary erythrocytes, and high CRN concentrations have been identified as negatively affecting the positive‐predictive value of urine NMP22.^19^, ^20^, ^21^ The negative results for urine NMP22 in previous studies were for standard values of ≤12 U/ml. NMP22 offered a better examination than expected, even in BD patients with hematuria or urinary inflammation in the present study.

The sensitivity of urine BTA was 74.6% and the positive‐predictive value of BTA was 40.7% in the present study, representing a major obstacle to using this as a tumor marker for screening examination in patients with NMIBC. Urine BTA has not been used in routine clinical examinations for patients with NMIBC.

Urine cytology is a common examination for patients with urothelial cancers. The problem of urine cytology is the significant individual variation seen among observers, and criteria for positive or negative status remain ambiguous. The Paris System Working Group, organized at the 2013 International Congress of Cytology proposed morphological criteria urging cytopathologists worldwide to categorize urine specimens.^22^, ^23^, ^24^ As a concept of The Paris System, urine cytology has been recognized as a useful diagnostic tool only for high‐grade urothelial carcinoma, because the accuracy of urine cytology for low‐grade urothelial carcinoma is generally low. We chose to classify urine cytology into 7 traditional categories, and did not apply recent criteria for urine cytology (including The Paris System) in the present study. The positive ratio of urine cytology was only 11.4% in patients with NMIBC (class 4, 9.8%; class 5, 1.6%; Figure 1D) and the AUC for low‐grade NMIBC was only 0.533, when classes 1‐3b were categorized as negative, and classes 4 and 5 as positive (Figure S3). These data for urine cytology indicated the unsuitability of this method in common screening for low‐grade NMIBC.

Most tumor markers generally offer high accuracy for high‐grade cancers and reduced accuracy for low‐grade cancers. Patients with high‐grade NMIBC can be detected with existing examinations, as in the present data for urine NMP22, BTA, and cytology. Many low‐grade NMIBCs yield negative results from these examinations.^25^ Urologists therefore require novel markers to detect low‐grade NMIBC. Our data demonstrated that the AUC for urine Ln‐γ2m/uCRN in patients with low‐grade NMIBC (0.725) was equivalent to that for high‐grade NMIBC (0.737). In contrast, the AUC for urine NMP22 in patients with low‐grade NMIBC (0.696) was lower than that for high‐grade NMIBC (0.795) (Figure 3). No significant differences were seen in these AUCs, whereas differences in the shapes of the ROC curves for urine Ln‐γ2m/uCRN and NMP22 suggested that measurement of urine Ln‐γ2m/uCRN might allow diagnosis of low‐grade NMIBC equivalent to that of high‐grade NMIBC. We identified immunohistochemical staining of Ln‐γ2 in the cytoplasm of low‐grade NMIBC (Figure S1). These findings might support the measurement of urine Ln‐γ2m/uCRN as a useful test even for patients with low‐grade NMIBC.

The value of urine Ln‐γ2m/uCRN in patients with BD was significantly higher than that in HD, whereas the value of urine NMP22 was not (Figure 1). In addition, the AUC of urine Ln‐γ2m/uCRN was significantly higher than that of NMP22 when NMIBC was compared with HD (but not to HD and BD combined) (Figure 2). These data suggested that the measurement of urine Ln‐γ2m/uCRN may contribute to distinguishing between healthy individuals and abnormal populations including BD and NMIBC in primary healthcare examinations. Measurement of urine NMP22 may contribute to distinguishing between non‐malignant populations including BD and NMIBC under secondary investigations.

One limitation is that a single urine marker alone cannot be used to definitively diagnose NMIBC. The correlation coefficients of each biomarker were all less than 0.5, indicating that the examinations were independent of each other (Figure S4). Given this and the data from DCA in Figure 4, combined examination with urine Ln‐γ2m/uCRN and NMP22 may compensate for the individual weaknesses of each test and may provide a new standard diagnostic procedure for NMIBC.

The sensitivity and specificity of cystoscopy have been high, reported as 68%–100% and 57%–97%, respectively.^26^ Cystoscopy is currently considered the ‘gold standard’ for diagnosing new or recurrent cases of bladder cancer. However, even the insertion of flexible fibers is physically and mentally invasive for patients. Measurement of urine Ln‐γ2m/uCRN may contribute to patients with abnormal urine findings under conditions such as health examinations that include many healthy individuals and the decision of whether the patient should undergo further, more‐invasive examinations such as cystoscopy in the setting of screening, and may also reduce the number of cystoscopies required after transurethral resection of bladder tumor.

Novel biological findings have recently been reported for Ln‐γ2m. Koshikawa et al. identified a fusion gene, LAMC2‐NR6A1, producing a short form of Ln‐γ2 (Lm‐γ2F), that possessed similar bioactivity for EGFR phosphorylation to that of the EGF ligand in ovarian cancer.^27^ Bladder cancer may also result in the presence of Lm‐γ2F in urine. The measurement of Ln‐γ2 by our chemiluminescent immunoassay procedure in the present study might have detected Lm‐γ2F in the urine of patients with NMIBC. Lm‐γ2F may play a crucial biological role and may be a novel molecular target for BC diagnostics and therapy.

In conclusion, the present investigation and our previous findings suggest that urine Ln‐γ2m/uCRN potentially represents a very useful novel biomarker for NMIBC, even in patients with low‐grade tumors and may contribute to populations including HD under primary healthcare examinations. The combined measurement of urine Ln‐γ2m/uCRN and NMP22 may be even more potent. Large‐scale prospective clinical research is required to confirm whether measurement of urine Ln‐γ2m/uCRN can contribute to the prognosis of patients with abnormal urine findings in health examinations. Cases of apparent BD and HD showing high expression of urine Ln‐γ2m in the present warrant stringent follow‐up for longer periods to identify whether they develop NMIBC in the future.

## AUTHOR CONTRIBUTIONS

T. Karashima contributed to the concept and drafted the manuscript. S. Umemoto collected specimens and cared for the patients. T. Kishida collected specimens and drafted the manuscript. K. Osaka collected specimens and cared for the patients. M. Nakagawa technically supported the measurement of Ln‐γ2m. E. Yoshida established the method for measuring Ln‐γ2m. T. Yoshimura established the method for measuring Ln‐γ2m. M. Sakaguchi managed and analyzed the data. H. Nishimoto managed the data. M. Tai managed the data. K. Inoue contributed to the concept and design. M. Seiki; contributed to the concept and design. N. Koshikawa contributed to the concept and design, and approved the final version of the manuscript. T. Shuin contributed to the concept and design, and approved the final version of the manuscript. All authors read and approved the final manuscript.

## CONFLICTS OF INTEREST

T. Yoshimura, E. Yoshida and M. Nakagawa are employees of Abbott Japan LLC. K. Inoue received patent royalties and research funds from SBI Pharmaceuticals Co., Ltd., honoraria from Chugai Pharmaceutical Co., Ltd., and endowments from Astellas Pharma Inc., Takeda Pharmaceutical Co., Ltd., MC Medical Inc. and Ono Pharmaceutical Co., Ltd. M. Seiki and N. Koshikawa received research funds from Tokyo University. N. Koshikawa and T. Shuin received research funds from Abbott Japan LLC. None of the other authors have any potential conflicts of interest.

## ETHICS STATEMENT

The study protocol was approved by our institutional review board (Kochi Medical School Hospital: 27–63; Kanagawa Cancer Center: 27–21).

## CONSENT TO PARTICIPATE

All patients and healthy donors provided written informed consent.

## CONSENT FOR PUBLICATION

All patients and healthy donors consented to publication.

## Supporting information


Appendix S1

Figure S1

Figure S2

Figure S3

Figure S4

## Data Availability

Requests for the study materials and dataset used to support the conclusions of this article should be directed to the corresponding author.
