# Supplementary material for: Clinical evaluation of urine laminin‐γ2 monomer as a potent biomarker for non‐muscle invasive bladder cancer
Source: Cancer Med. 2022 Aug 4;12(3):2453–62. doi: 10.1002/cam4.5087 (PMC9939167; doi:10.1002/cam4.5087)
Supplement: Supplementary file 1 — Appendix S1 [file CAM4-12-2453-s001.docx]

**Eligibility Criteria**

**1. Inclusion Criteria**

1) Signed Written Informed Consent

2) Age and Reproductive Status

a) Males and females

b) Age 20–90 years old

3) Type of Participant and Target Disease Characteristics

a) Patients with urothelial cancer in the bladder

b) Patients treated with transurethral resection of bladder tumor

**2. Exclusion Criteria**

1) Patients planning to undergo radical cystectomy

2) Patients less than 6 months after final therapy for bladder cancer

3) Patients with a history of prior active malignancy other than bladder cancer within the last 5 years

4) Patients with suspected malignancy other than bladder cancer

5) Patients with acute inflammatory disease

6) Patients with severe hepatic or renal dysfunction

7) Written informed consent not signed

8) Patients deemed by the physician in charge as unsuitable for inclusion in the study
