# Supplementary figures and images for: Clinical evaluation of urine laminin‐γ2 monomer as a potent biomarker for non‐muscle invasive bladder cancer
Source: Cancer Med. 2022 Aug 4;12(3):2453–62. doi: 10.1002/cam4.5087 (PMC9939167; doi:10.1002/cam4.5087)

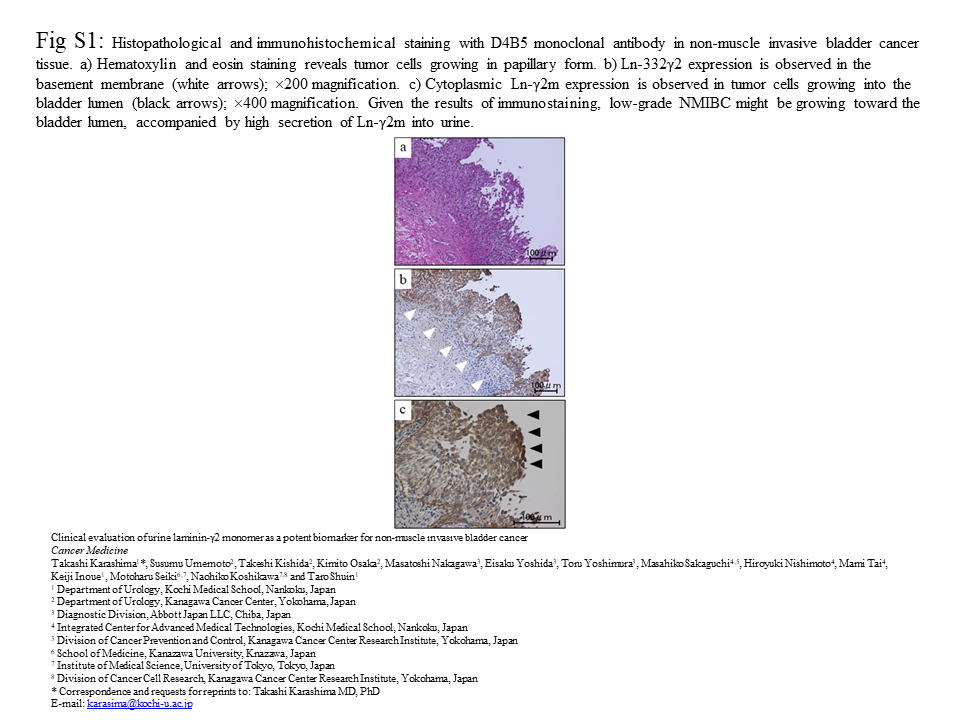

Supplement: Supplementary file 2 — Figure S1 [file CAM4-12-2453-s002.tif]
