# Supplementary material for: Clinical evaluation of urine laminin‐γ2 monomer as a potent biomarker for non‐muscle invasive bladder cancer
Source: Cancer Med. 2022 Aug 4;12(3):2453–62. doi: 10.1002/cam4.5087 (PMC9939167; doi:10.1002/cam4.5087)
Supplement: Supplementary file 3 — Figure S2 [file CAM4-12-2453-s005.pdf]

Fig S2: ROC curve and AUC for the combination of urine Ln-r2/uCRN and NMP22 in patients with NMIBC. The AUC for combined examination using urine Ln-γ2m/uCRN and NMP22 was 0.796, whereas the AUCs for the single examinations were 0.733 and 0.759, respectively *(left)*. In patients with low-grade NMIBC, the AUCs were 0.752, 0.725 and 0.696, respectively *(right)*.

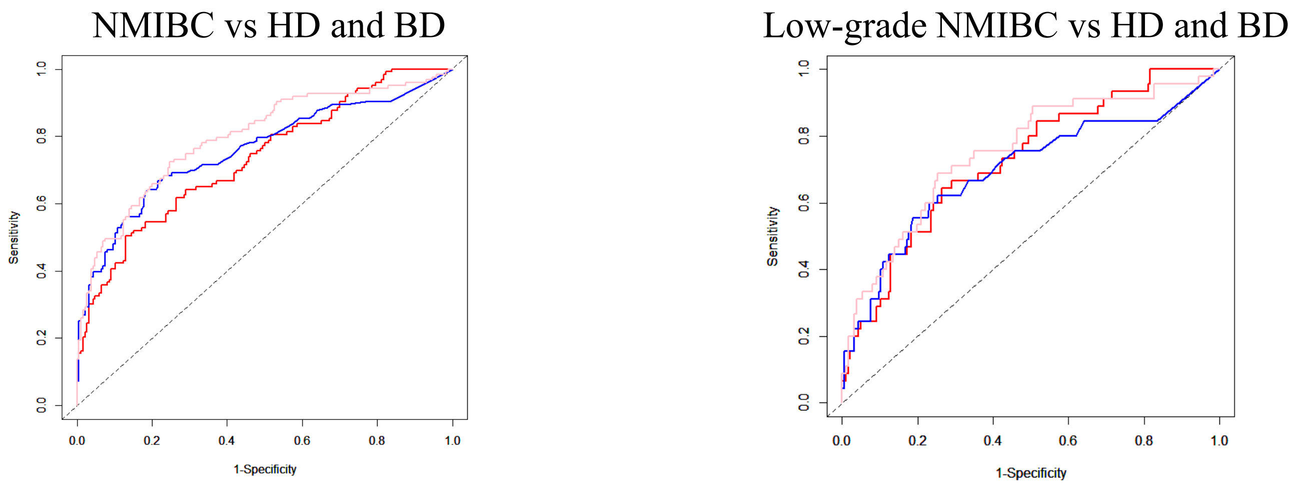

Examination	AUC (95%CI) NMIBC vs HD and BD	AUC (95%CI) Low-grade NMIBC vs HD and BD
Urine Ln-r2/uCRN+NMP22	0.796 (0.743-0.849)	0.752 (0.668-0.836)
Urine Ln-r2/uCRN	0.733 (0.676-0.79)	0.725 (0.644-0.807)
Urine NMP22	0.759 (0.7-0.817)	0.696 (0.793-0.86)

ROC, receiver operating characteristic; AUC, area under the ROC curve; HD, healthy donors; BD, benign genitourinary disease; NMIBC, non-muscle invasive bladder cancer; CI, confidence interval.

Clinical evaluation of urine laminin-γ2 monomer as a potent biomarker for non-muscle invasive bladder cancer
Cancer Medicine
Takashi Karashima[1]*, Susumu Umemoto[2], Takeshi Kishida[2], Kimito Osaka[2], Masatoshi Nakagawa[3], Eisaku Yoshida[3], Toru Yoshimura[3], Masahiko Sakaguchi[4,5], Hiroyuki Nishimoto[4], Mami Tai[4], Keiji Inoue[1], Motoharu Seiki[6,7], Naohiko Koshikawa[7,8] and Taro Shuin[1]
[1] Department of Urology, Kochi Medical School, Nankoku, Japan
[2] Department of Urology, Kanagawa Cancer Center, Yokohama, Japan
[3] Diagnostic Division, Abbott Japan LLC, Chiba, Japan
[4] Integrated Center for Advanced Medical Technologies, Kochi Medical School, Nankoku, Japan
[5] Division of Cancer Prevention and Control, Kanagawa Cancer Center Research Institute, Yokohama, Japan
[6] School of Medicine, Kanazawa University, Knazawa, Japan
[7] Institute of Medical Science, University of Tokyo, Tokyo, Japan
[8] Division of Cancer Cell Research, Kanagawa Cancer Center Research Institute, Yokohama, Japan
* Correspondence and requests for reprints to: Takashi Karashima MD, PhD
E-mail: karasima@kochi-u.ac.jp
