# Supplementary material for: Clinical evaluation of urine laminin‐γ2 monomer as a potent biomarker for non‐muscle invasive bladder cancer
Source: Cancer Med. 2022 Aug 4;12(3):2453–62. doi: 10.1002/cam4.5087 (PMC9939167; doi:10.1002/cam4.5087)
Supplement: Supplementary file 4 — Figure S3 [file CAM4-12-2453-s004.pdf]

Fig S3: ROC curve and AUC for each examination in patients with low-grade NMIBC. The AUCs for urine Ln-r2/uCRN, NMP22, BTA and cytology were 0.725, 0.696 0.599 and 0.533, respectively.

Low-grade NMIBC vs HD and BD

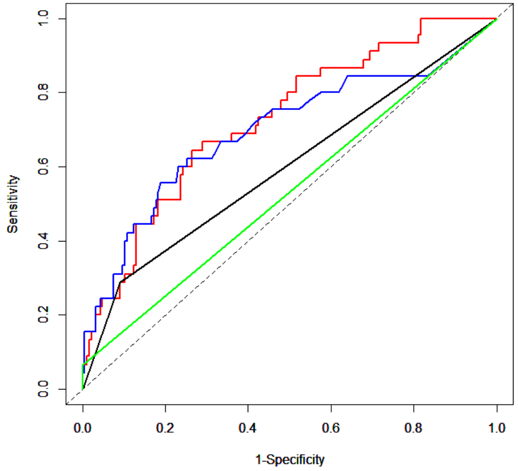

Examination	AUC (95%CI)
Urine Ln-r2/uCRN	0.725 (0.644-0.807)
Urine NMP22	0.696 (0.599-0.793)
Urine BTA	0.599 (0.529-0.669)
Urine cytology	0.533 (0.497-0.57)

ROC, receiver operating characteristic; AUC, area under the ROC curve; HD, healthy donors; BD, benign genitourinary disease; NMIBC, non-muscle invasive bladder cancer; CI, confidence interval.

Clinical evaluation of urine laminin-γ2 monomer as a potent biomarker for non-muscle invasive bladder cancer

Cancer Medicine

Takashi Karashima[1]*, Susumu Umemoto[2], Takeshi Kishida[2], Kimito Osaka[2], Masatoshi Nakagawa[3], Eisaku Yoshida[3], Toru Yoshimura[3], Masahiko Sakaguchi[4,5], Hiroyuki Nishimoto[4], Mami Tai[4], Keiji Inoue[1], Motoharu Seiki[6,7], Naohiko Koshikawa[7,8] and Taro Shuin[1]
[1] Department of Urology, Kochi Medical School, Nankoku, Japan
[2] Department of Urology, Kanagawa Cancer Center, Yokohama, Japan
[3] Diagnostic Division, Abbott Japan LLC, Chiba, Japan
[4] Integrated Center for Advanced Medical Technologies, Kochi Medical School, Nankoku, Japan
[5] Division of Cancer Prevention and Control, Kanagawa Cancer Center Research Institute, Yokohama, Japan
[6] School of Medicine, Kanazawa University, Knazawa, Japan
[7] Institute of Medical Science, University of Tokyo, Tokyo, Japan
[8] Division of Cancer Cell Research, Kanagawa Cancer Center Research Institute, Yokohama, Japan
* Correspondence and requests for reprints to: Takashi Karashima MD, PhD
E-mail: karasima@kochi-u.ac.jp
