# Supplementary material for: Clinical evaluation of urine laminin‐γ2 monomer as a potent biomarker for non‐muscle invasive bladder cancer
Source: Cancer Med. 2022 Aug 4;12(3):2453–62. doi: 10.1002/cam4.5087 (PMC9939167; doi:10.1002/cam4.5087)
Supplement: Supplementary file 5 — Figure S4 [file CAM4-12-2453-s003.pdf]

Fig S4: Correlations of urine Ln-γ2m/uCRN, NMP22 and BTA. All correlation coefficients were low, with no correlations evident between any biomarkers.

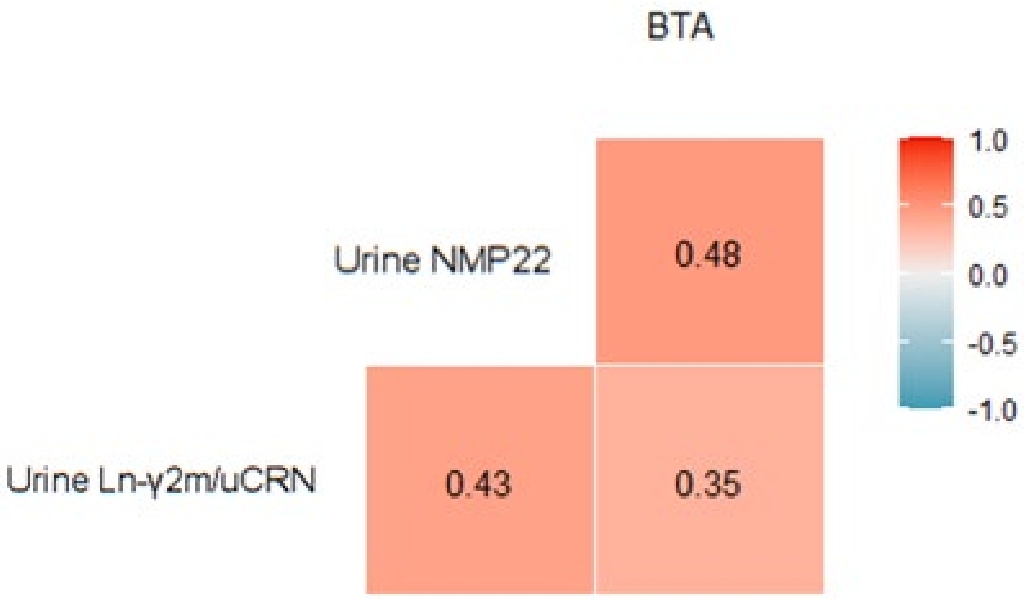

Clinical evaluation of urine laminin-γ2 monomer as a potent biomarker for non-muscle invasive bladder cancer
Cancer Medicine
Takashi Karashima[1]*, Susumu Umemoto[2], Takeshi Kishida[2], Kimito Osaka[2], Masatoshi Nakagawa[3], Eisaku Yoshida[3], Toru Yoshimura[3], Masahiko Sakaguchi[4,5], Hiroyuki Nishimoto[4], Mami Tai[4], Keiji Inoue[1], Motoharu Seiki[6,7], Naohiko Koshikawa[7,8] and Taro Shuin[1]
[1] Department of Urology, Kochi Medical School, Nankoku, Japan
[2] Department of Urology, Kanagawa Cancer Center, Yokohama, Japan
[3] Diagnostic Division, Abbott Japan LLC, Chiba, Japan
[4] Integrated Center for Advanced Medical Technologies, Kochi Medical School, Nankoku, Japan
[5] Division of Cancer Prevention and Control, Kanagawa Cancer Center Research Institute, Yokohama, Japan
[6] School of Medicine, Kanazawa University, Knazawa, Japan
[7] Institute of Medical Science, University of Tokyo, Tokyo, Japan
[8] Division of Cancer Cell Research, Kanagawa Cancer Center Research Institute, Yokohama, Japan
* Correspondence and requests for reprints to: Takashi Karashima MD, PhD
E-mail: karasima@kochi-u.ac.jp
